# Supplementary material for: A modularity analysis helps improving the structure of the International Code of Zoological Nomenclature
Source: PeerJ. 2021 Feb 23;9:e10815. doi: 10.7717/peerj.10815 (PMC7908869; doi:10.7717/peerj.10815)
Supplement: Supplemental Information 3 [file peerj-09-10815-s003.pdf]

Gmail **Evangelos Vlachos <evlacho@gmail.com>**

Permission to use the text of the Code for a network analysis

6 messages

Evangelos Vlachos <evlacho@gmail.com> 8 November 2020 at 19:30
To: "Secretary, ICZN" <iczn@nus.edu.sg>, Thomas Pape <tpape@snm.ku.dk>

Dear Secretary, and dear Prof. Pape,

I hope that this email finds you well, especially amidst this global pandemic.

If you remember, I contacted you some time ago (July 2018), asking permission to use the text of the Code for a network analysis, which you kindly granted to me. The result of that scientific project has been published here, and additional information is hosted on my personal institutional website, where I always acknowledge and the International Trust for ZooNom for permission.

Since then, I have been working more with this methodology and I almost finished the next and important analysis based on the network of the Code: a modularity analysis. I use the network to recognize conceptual modules that group together the various parts of the Code according to their connections, forming an hierarchical structure. In other words, I seek to find the conceptual structure of the Code according to the network, and also identify potential and possible conflicts with the current structure of the Code.

With this email, I would like to kindly ask to renew, if possible and/or necessary, the permission to use the text of the Code in the form of a network for my analysis. My idea to try to publish this second paper in an open access journal, starting with PeerJ as the original paper; that way, taxonomists will have the opportunity to access the work.

I also want to create some kind of poster depicting the recovered conceptual structure (see attached for an early example; not final, I will double-check everything). My analysis has recovered some 65 different submodules, grouped in 10, larger, modules, and using the network I have been able to create this short of diagram that is reminiscent of the periodic table of elements. My intention with that "poster" is that users should be able to download and share with (probably) a CC-BY-NC license (for non-commercial purposes), unless otherwise requested by the Commission. This figure is the complex original network published in the original work, but modularized, simplified and organized.

This modularity analysis is an applied product on the theoretical network and my most important goal, because I firmly believe that it lays the path for a web and/or mobile application and could help in using, teaching, and promoting the Code, nomenclature, and taxonomy to a wider public and to younger generations of taxonomists. I hope that perhaps now it becomes more clear to you where I was going with this new methodology and which is my vision of the Code.

I think that it would be great if you could confirm to me that I can use the text of the Code (herein expressed as the modules derived by my analysis) to create this kind of poster. I think that this is, actually, a quite derivative work that might not require permission, but I prefer to ask you first.

Of course, please feel free to share this, if necessary, with other members of the Commission.

Thank you very much in advance for considering this request. Also, I hope that you will like it and any constructive criticism is more than welcome.

Sincerely,
Evan Vlachos

Fig-3_Periodic Table.pdf
618K

Thomas Pape <tpape@snm.ku.dk> 8 November 2020 at 19:42
To: Evangelos Vlachos <evlacho@gmail.com>
Cc: "Secretary, ICZN" <iczn@nus.edu.sg>

Dear Evan,

Thank you for your mail. This is an entirely new way of looking at nomenclatural legislation, so let me spend a few days trying to digest these complicated issues. I will get back as soon as possible.

Regards,

Thomas

[Quoted text hidden]

Evangelos Vlachos <evlacho@gmail.com> 9 November 2020 at 09:28
To: Thomas Pape <tpape@snm.ku.dk>
Cc: "Secretary, ICZN" <iczn@nus.edu.sg>

Dear Thomas, thank you very much, deeply appreciated. I can always offer a presentation or demonstration of this method if required!

All the best,

Evan
[Quoted text hidden]

Secretary, ICZN <iczn@nus.edu.sg> 9 November 2020 at 13:02
To: Evangelos Vlachos <evlacho@gmail.com>, Thomas Pape <tpape@snm.ku.dk>
Cc: "icznsg@gmail.com" <icznsg@gmail.com>

Dear Evan,

Thank you very much for sharing this with us. I will archive a digital copy of this for the Commission's reference.

Best Wishes,

Gwynne

From: Evangelos Vlachos <evlacho@gmail.com>
Sent: Sunday, November 8, 2020 5:31 PM
To: Secretary, ICZN <iczn@nus.edu.sg>; Thomas Pape <tpape@snm.ku.dk>
Subject: Permission to use the text of the Code for a network analysis

- External Email -

[Quoted text hidden]

Thomas Pape <tpape@snm.ku.dk> 12 November 2020 at 20:13
To: Evangelos Vlachos <evlacho@gmail.com>

Cc: "Secretary, ICZN" <iczn@nus.edu.sg>

Dear Evan,

You can use the text of the Code for your modularity analysis. I find your 'periodic table' very interesting, and it makes sense to see the groupings. Please keep me updated on your studies based on our nomenclatural Code.

Actually, I have tried to attend some of your tutorials, but network-thinking is an entirely new field for me.

Regards,

Thomas

From: Evangelos Vlachos <evlacho@gmail.com>
Sent: 8. november 2020 23:31
To: Secretary, ICZN <iczn@nus.edu.sg>; Thomas Pape <tpape@snm.ku.dk>

[Quoted text hidden]

[Quoted text hidden]

Evangelos Vlachos <evlacho@gmail.com> 13 November 2020 at 09:47
To: Thomas Pape <tpape@snm.ku.dk>
Cc: "Secretary, ICZN" <iczn@nus.edu.sg>

Dear Thomas,

thank you very much for granting permission to use the text of the Code. I am working now on the second manuscript of the ICZN Network or Neticon, planning to focus on the modularity analysis and reach the conclusion of this "periodic table".

The most interesting result of the analysis, and given all the attacks that the structure of the Code has received is the following: two thirds of the Code have excellent or very good structure, meaning that the conceptual network models that they are found correspond very well to the current organization and structure.

Only a third of the Code has a conceptual structure that is in conflict with the current structure, and the main problem is the fact that provisions dealing with different aspects (e.g., validity, homonymy, typification) of the same group (family-, genus-, species-group) are placed in different Articles and/or Chapters. Instead if all provisions for the same group were in the same place, it would make more sense. Also, it seems that the flow would be better if the order of presentation of the different rank would be inverted, first the species-group, and then genus and family.

Of course I am not proposing radical changes in the Code, but rather suggest that these observations could be used to improve its use and teaching.

Sincerely,

Evan
[Quoted text hidden]
